# Enhancement of Silymarin Anti-fibrotic Effects by Complexation With Hydroxypropyl (HPBCD) and Randomly Methylated (RAMEB) β-Cyclodextrins in a Mouse Model of Liver Fibrosis

**DOI:** 10.3389/fphar.2018.00883

**Published:** 2018-08-13

**Authors:** Sami Gharbia, Cornel Balta, Hildegard Herman, Marcel Rosu, Judit Váradi, Ildikó Bácskay, Miklós Vecsernyés, Szilvia Gyöngyösi, Ferenc Fenyvesi, Sorina N. Voicu, Miruna S. Stan, Roxana E. Cristian, Anca Dinischiotu, Anca Hermenean

**Affiliations:** ^1^The Institute of Life Sciences, Vasile Goldis Western University of Arad, Arad, Romania; ^2^Department of Pharmaceutical Technology, Faculty of Pharmacy, University of Debrecen, Debrecen, Hungary; ^3^Department of Solid State Physics, University of Debrecen, Debrecen, Hungary; ^4^Department of Biochemistry and Molecular Biology, The Faculty of Biology, University of Bucharest, Bucharest, Romania; ^5^Department of Histology, Faculty of Medicine, Vasile Goldis Western University of Arad, Arad, Romania

**Keywords:** silymarin, HPBCD, RAMEB, liver fibrosis, ECM, collagen

## Abstract

Silymarin (Sy) shows limited water solubility and poor oral bioavailability. Water-soluble hydroxypropyl (HPBCD) and randomly methylated (RAMEB) β-cyclodextrins were designed to enhance anti-fibrotic efficiency of silymarin in CCl_4_-induced liver fibrosis in mice. Experimental fibrosis was induced by intraperitoneal injection with 2 ml/kg CCl_4_ (20% v/v) twice a week, for 7 weeks. Mice were orally treated with 50 mg/kg of Sy-HPBCD, Sy-RAMEB and free silymarin. For assessment of the spontaneous reversion of fibrosis, CCl_4_ treated animals were investigated after 2 weeks of recovery time. The CCl_4_ administration increased hepatic oxidative stress, augmented the expression of transforming growth factor-β1 (TGF-β1) and Smad 2/3, and decreased Smad 7 expression. Furthermore, increased α-smooth muscle actin (α-SMA) expression indicated activation of hepatic stellate cells (HSCs), while up-regulation of collagen I (Col I) and matrix metalloproteinases (MMPs) expression led to an altered extracellular matrix enriched in collagen, confirmed as well by trichrome staining and electron microscopy analysis. Treatment with Sy-HPBCD and Sy-RAMEB significantly reduced liver injury, attenuating oxidative stress, restoring antioxidant balance in the hepatic tissue, and significantly decreasing collagen deposits in the liver. The levels of pro-fibrogenic markers’ expression were also significantly down-regulated, whereas in the group for spontaneous regression of fibrosis, they remained significantly higher, even at 2 weeks after CCl_4_ administration was discontinued. The recovery was significantly lower for free silymarin group compared to silymarin/β cyclodextrins co-treatments. Sy-HPBCD was found to be the most potent anti-fibrotic complex. We demonstrated that Sy-HPBCD and Sy-RAMEB complexes decreased extracellular matrix accumulation by inhibiting HSC activation and diminished the oxidative damage. This might occur via the inhibition of TGF-β1/Smad signal transduction and MMP/tissue inhibitor of MMPs (TIMP) rebalance, by blocking the synthesis of Col I and decreasing collagen deposition. These results suggest that complexation of silymarin with HPBCD or RAMEB represent viable options for the its oral delivery, of the flavonoid as a potential therapeutic entity candidate, with applications in the treatment of liver fibrosis.

## Introduction

Hepatic fibrosis is a pathological consequence of chronic liver diseases and results in excessive scar tissue, due to an incorrect wound healing response to liver injury. This pathology is characterized by necrosis and/or apoptosis of parenchymal cells and their replacement with an altered extracellular matrix (ECM) enriched in types I and III fibrillar collagens (Mallat and Lotersztajn, 2013). Accumulation of ECM proteins changes the liver architecture and ultimately leads to cirrhosis, a condition defined by an abnormal parenchymal structure, with fibrotic septa surrounding regenerating nodules (Friedman, 2003) and pathologic angiogenesis (Bocca et al., 2015). Findings in both human studies and animal models revealed that development of liver fibrosis is a dynamic process that can be modulated by arresting progression and/or promoting resolution (Trautwein et al., 2015).

Several studies have been conducted to find the main herbal formulations and plant bioactive molecules designed for liver fibrosis therapy (Hermenean et al., 2016). Most of the natural anti-fibrotic compounds, i.e., chrysin (Balta et al., 2015), caffeine (Wang et al., 2015), morin (Heeba and Mahmoud, 2014; MadanKumar et al., 2014, 2015), mistletoe alkaloid fractions (Jiang et al., 2014) or Chinese herbal formula (Lin et al., 2011, 2012), have directly inactivated hepatic stellate cells (HSC), myofibroblasts and the ECM. Some “indirect antifibrotics”, such as *Amomum xanthoides* extract (Wang et al., 2011) and *Terminalia bellerica* aerial parts ethyl acetate extract (Rashed et al., 2014), addressed to other pathways.

Silymarin, a flavonoid complex a mixture of flavonolignans and taxifolin, is extracted from the seeds of milk thistle (*Silybum marianum* L.). The active constituents of silymarin are: silibinin, isosilybinin, silydianin, and silychristin, of which silibinin is the major and most active component, representing about 60–70% of flavonoids (Saller et al., 2001).

Silymarin and silibinin show the ability to protect mammals’ liver against hepatotoxicity induced by ethanol (Zhang et al., 2013), carbon tetrachloride (Yadav et al., 2008; Salam et al., 2009; Shaker et al., 2010), cisplatin (Mansour et al., 2006; Abdel-Rahman and Abdel-Hady, 2013), arsenic (Jain et al., 2011; Muthumani and Prabu, 2012), anti-tuberculosis drugs (Eminzade et al., 2008), thioacetamide (Ghosh et al., 2016) and acetaminophen (Avizeh et al., 2010), as it has been previously shown. Moreover, silibinin have showed efficacy to alleviate non-alcoholic fatty liver disease, through nicotinamide adenine dinucleotide (NAD+) level restoration (Salomone et al., 2017).

One of the major limitations of silymarin is the poor water solubility and oral-bioavailability. Its oral absorption is only about 23–47% (Ghosh et al., 2010), generating an oral bioavailability of 0.73% (Wu et al., 2007). This poor bioavailability results from the instability in gastric environment (Blumenthal et al., 2000), poor intestinal absorption (Loguercio and Festi, 2011) and poor water solubility (Blumenthal et al., 2000). This underlines that very high amount of Sylimarin are needed in order to achieve biological activities. In this sense, Clichici et al. (2016) demonstrated that at least 200 mg/kg are necessary to reduce the extent of the experimental fibrosis to the liver. However, concerns about the administration of high doses of silymarin remain, while some results show that it has a toxic dose of >1.44 g per week (Wu et al., 2011).

Some pharmaceutical formulations, such as nanostructured lipid carriers (NLCs) (Jia et al., 2010), liposomes (El-Samaligy et al., 2006; Elmowafy et al., 2013; Kumar et al., 2014), nanoemulsions (Parveen et al., 2011), solid lipid nanoparticles (Cengiz et al., 2015), gold nanoparticles (Kabir et al., 2014), alginate-poly (lactic-co-glycolic acid) nano/micro hydrogel matrices (El-Sherbiny et al., 2011), self-nanoemulsifying drug delivery systems (Iosio et al., 2011; Chen et al., 2015), have been used to increase the aqueous solubility of silymarin. Among these, gold nanoparticles were designed to ameliorate liver fibrosis, and Au-silymarin conjugates were administered for up to 14 weeks without any noticeable side effects in the morphology of heart, kidney and lungs of the animals (Kabir et al., 2014).

Cyclodextrins (CDs) are a cyclic oligosaccharides family useful for drug delivery of poor water-soluble natural compounds in order to enhance their solubility, bioavailability, and stability when are orally administered (Suvarna et al., 2017).

In this study, silymarin – hydroxypropyl-β-cyclodextrin (Sy-HPBCD) and silymarin – randomly methylated-β-cyclodextrin (Sy-RAMEB) complexes were developed in order to improve silymarin anti-fibrotic activity at the lowest therapeutical dose, by increasing their potential solubilization and to prevent their metabolic degradation within the gastrointestinal tract after oral administration. To our knowledge, there are no thorough studies so far, reporting Sy-HPβCD and Sy-RAMEB anti-fibrotic activity in animal models of liver fibrosis. We proposed that Sy-HPBCD and/or Sy-RAMEB could attenuate the progression of liver fibrosis better than silymarin itself, by reducing oxidative stress and expression of inflammatory and pro-fibrogenic markers in the progression of CCl_4_-induced liver fibrosis.

## Materials and Methods

### Reagents and Antibodies

All chemicals and solvents used in the study were of analytical grade.

2-Hydroxypropyl)-beta-cyclodextrin (HPBCD) and methyl-beta-cyclodextrin (RAMEB) were purchased from Cyclolab Ltd. (Budapest, Hungary). Silymarin 98% and carboxymethyl cellulose were purchased from Sigma-Aldrich (Germany). Rabbit polyclonal TGF-β1 (sc-146), rabbit polyclonal Smad 2/3 (sc-8332), mouse monoclonal α-SMA (sc-53142), rabbit polyclonal NF-κB p65 (sc-109) antibodies were supplied from Santa Cruz Biotechnology (Santa Cruz, CA, United States), rabbit polyclonal Anti-Col-I antibody (ab34710) from Abcam (United States) and mouse monoclonal Anti-MMP-1 antibody (NBP2-22123) from NovusBio (Novus, United States). Novocastra kit for immunohistochemistry was purchased from Leica Microsystems (Germany).

### Preparation of Silymarin-Cyclodextrin Physical Mixtures

The required amount of silymarin and cyclodextrins (RAMEB and HPBCD) were measured out and mixed thoroughly in mortar without using solvent. The prepared physical mixtures (PM) were used in solubility and SEM studies.

### Preparation of Silymarin-Cyclodextrin Complexes

Silymarin-cyclodextrin complexes were produced with HPBCD and RAMEB. Complexes were prepared by kneading method. Briefly, silymarin was dissolved in 96% ethanol and mixed with cyclodextrin in a mortar to obtain a mixture with 1:20 silymarin: cyclodextrin mass ratio (1:6 molar ratio). The mixture was dried at 30°C with continuous mixing. After drying the products were ground and mixed thoroughly again. At first Silymarin was dissolved in 96% ethanol to obtain a concentrated (8 mg/ml) Silymarin solution. Cyclodextrins were measured into mortars and mixed with the required amount of Silymarin solution to obtain a mixture with 1:20 silymarin: cyclodextrin mass ratio (1:6 molar ratio). As both RAMEB and HPBCD are soluble in ethanol cyclodextrins were also dissolved in the ethanolic Silymarin solution at this step. The ethanol was evaporated from the solutions at 30°C with continuous mixing. During evaporation the solutions containing cyclodextrins and silymarin were concentrated and viscous pastes were obtained, which were further kneaded and dried continuously. After perfect drying the products were ground and mixed thoroughly again.

### Phase-Solubility Study

Aliquots of 100 mg silymarin were measured into 12-well plates. Cyclodextrin solutions were prepared in the concentration range of 0–76 mM by Milli-Q water. A volume of 2 ml of cyclodextrin solutions was measured into the wells and mixed with silymarin powder. The mixtures were shaken for 48 h at 22°C. After the incubation, 300 μl supernatant was removed from each samples and analyzed by High Performance Liquid Chromatography (HPLC) to determine the concentration of the dissolved silymarin. Using the concentration data, phase-solubility curves were plotted for the characterization of silymarin complexation by cyclodextrins (Higuchi and Connors, 1965; Loftsson et al., 2005a,b; Jambhekar and Breen, 2016).

The 1:1 molar ratio of silymarin:CD inclusion complexes was assumed. The stability constants (*K*_1:1_) were calculated from the slope of the phase-solubility diagrams according to the equation:

$${\text{K}}_{\text{1}:\text{1}}\;\text{=}\;\text{slope/}[{\text{S}}_{\text{0}}(\text{1-slope})],$$

where *S*_0_ is the solubility of the silymarin components in the absence of cyclodextrins.

Complexation efficiencies were calculated from the slope of the phase-solubility diagrams according to the equation:

$$\text{CE}\;\text{=}\;S_{\text{0}}\cdot K_{\text{1}:\text{1}}\;\text{=}\;\text{slope/}(\text{1-slope}).$$

### HPLC Analysis

The samples were analyzed using a HPLC system Merck-Hitachi ELITE LaCrom consisting of a pump (L-2130), degasser, automated injector, column oven (L-2300) and a photodiode array detector (DAD). The column module was kept at 40°C, and the DAD was set to collect signals within the spectral range of 220–400 nm. The separation of silymarin components was performed on the Zorbax SB C-18 column (4.6 mm × 75 mm, 3.5 μm) (Agilent, Santa Clara, CA, United States). The injected volume of samples was 100 μl. A gradient elution system (flow rate of 0.8 ml min^-1^) was applied. The mobile phase A was water containing 0.1% (v/v) formic acid, and mobile phase B was methanol. The gradient condition was the following: at the beginning 80:20 (v/v) A and B, 0–80 min linear change to 50:50 (v/v) A:B. The analyses were performed with EZChrom Elite software^TM^ (Hitachi, Tokyo, Japan), which was also used for collecting and processing data.

### Scanning Electron Microscopy (SEM)

The morphology of solid particles of cyclodextrin, silymarin, silymarin-cyclodextrin physical mixtures and silymarin-cyclodextrin complexes were investigated by Hitachi S-4300 CFE SEM (Scanning Electron Microscope) using 15 kV accelerating voltage. The specimens were coated with gold for imaging.

### Animals and Experimental Design

Male CD1 mice from our breeding colony, weighting 26 ± 3 g (5–6 weeks old), were used for this study. The mice were fed with a standard rodent diet and were maintained at 12 h light/dark cycle at constant temperature and humidity. All experimental procedures were approved by the Ethical Committee of the “Vasile Goldis” Western University of Arad and certified by the National Sanitary Veterinary and Food Safety Authority of Romania.

**FIGURE 1:**
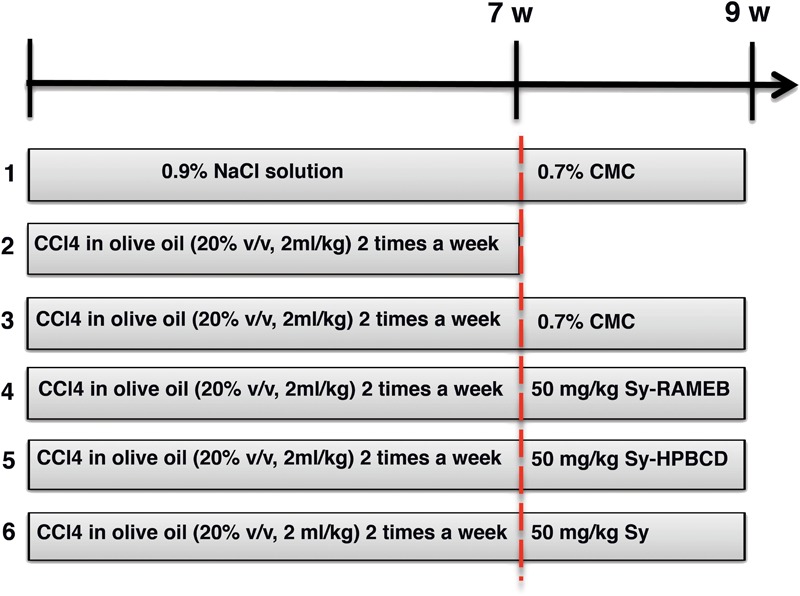
Schematic diagram of the experimental protocol.

Mice were divided into six groups of 10 (**Figure 1**). Group 1 (control group) was given orally 0.9% NaCl solution for 7 weeks, followed by 0.7% carboxymethyl cellulose (CMC) as for 2 weeks. Group 2 (CCl_4_ group) received CCl_4_ in olive oil (20% v/v, 2 ml/kg b.w.) 2 times a week for 7 weeks and were euthanatized 72 h after the last injection for the confirmation of liver fibrosis. Group 3 (CCl_4_ control group) received CCl_4_ in olive oil (20% v/v, 2 ml/kg b. w.) 2 times a week for 7 weeks, followed by 2 weeks of recovery for the fibrosis spontaneous resolution analysis. Group 4 (CCl_4_/Sy-RAMEB group) was given CCl_4_ as to groups 2 and 3, followed by 50 mg/kg b.w. Sy-RAMEB orally administration once a day for 2 weeks more. Group 5 (CCl_4_/Sy-HPBCD group) was given CCl_4_ as to groups 2 and 3, followed by 50 mg/kg b.w. Sy-HPBCD orally administration once a day for 2 weeks more. Group 6 (CCl_4_/Sy group) was given CCl_4_ as group to 2 and 3, followed by 50 mg/kg b.w. free silimarin orally administration once a day for 2 weeks more.

The dose of silymarin was selected based on previous investigations that used the lowest effective dose for liver fibrosis alleviation (Mourelle et al., 1989; Boigk et al., 1997).

At the end of the 9-week treatment (1–6 groups, except group 2), mice were euthanatized, liver biopsies were collected for biochemical, histological and electron microscopy evaluations. The remaining liver tissues were snap-frozen to extract the total RNA.

### Cell Lysate Preparation

Mice livers (0.1 g of tissue) were mixed with 1 ml of ice-cold 0.1 M TRIS-HCl – 5 mM EDTA buffer (pH 7.4) and homogenized for 2 min at 30 Hz using a ball mill (type MM 301, Retsch GmbH & Co, Haan, Germany). The samples were centrifuged at 10000 × *g* for 10 min at 4°C to obtain the supernatants which were further used for the biochemical assays. The protein content was measured using the Bradford reagent and bovine serum albumin as standard.

### Measurement of Lipid Peroxidation

The content of hepatic malondialdehyde (MDA) was measured in order to estimate lipid peroxidation. The fluorimetric technique previously described by Dinischiotu et al. (2013) was based on the reaction of MDA with thiobarbituric acid. Relative fluorescence units read at FP-6300 JASCO spectrofluorometer (λex = 520 nm; λem = 549 nm) were transformed to nmol MDA using 1,1,3,3-tetramethoxypropane as standard. The values were normalized to the protein concentration and shown as percentages of control.

### Assessment of Reduced Glutathione (GSH) Level

The cell lysates were deproteinized with 5% sulfosalicylic acid (Sigma-Aldrich, United States) and the GSH content was spectrophotometrically measured, using the commercial glutathione assay kit (Sigma-Aldrich), based on the reduction of 5,5′-dithiobis-2-nitrobenzoic acid (DTNB) into 5-thio-2-nitrobenzoic acid (TNB). The absorbance was recorded at 405 nm using the FlexStation 3 multi-mode microplate reader and the concentration was calculated in nmoles/mg protein, presented as a percentage of control.

### Determination of Advanced Oxidation Protein Products (AOPP) Concentration

The AOPP concentration was assessed as previously described (Petrache et al., 2012). A volume of 200 μL protein extract was incubated with 10 μL of 1.16 M potassium iodide and 20 μL of glacial acetic acid. The optical densities were read at 340 nm in a 96-well plate using the FlexStation 3 multi-mode microplate reader and the AOPP levels were calculated using a chloramine-T standard curve and reported to the protein concentration.

### Measurement of Carbonyl Groups Content

The concentration of carbonyl groups was assessed as described in a previous work (Petrache et al., 2012). Briefly, the diluted total protein extract was incubated with an equal volume of 10 mM 2,4-dinitrophenylhydrazine for 1 h at room temperature, and after that, 20% TCA was added in a volume equal to previous solution. The mixture was incubated for 30 min on ice and centrifuged for 3 min at 13,000 rpm at room temperature in order to obtain the pellet which was washed with ethanol: ethyl acetate (1:1) solution. Finally, the pellet was rendered soluble in 500 μL of 1 M NaOH, and the absorbance was read at 370 nm and reported to the protein concentration of each sample.

### Western Blot

The protein levels of MMPs and TIMP-1 were quantified by Western blotting using the protein supernatants collected as described above in the “Cell lysate preparation” section incubated with primary rabbit polyclonal antibodies anti-MMP-2 and anti-MMP-9 (dilution 1:250; Santa Cruz Biotechnology, Santa Cruz, CA, United States). Proteins were separated (40 μg/well) was done by sodium dodecyl sulfate-polyacrylamide gel electrophoresis (SDS/PAGE, 10% acrylamide) under reducing conditions and transferred onto 0.4 μm poly(vinylidene difluoride) membrane (Millipore) in a wet transfer system (Bio-Rad, Hercules, CA, United States). The next steps (membrane blocking, incubation antibodies and with chromogen solution) were performed using the WesternBreeze Chromogenic kit (Invitrogen) and the membranes were processed according to manufacturer’s instructions. The obtained bands were visualized with the Chemidoc MP system (Bio-Rad, Hercules, CA, United States) and quantified using ImageLab software (Bio-Rad, Hercules, CA, United States). Each sample tested was normalized to the expression corresponding to β-actin band used as a control of protein loading.

### Gelatin Zymography

The enzymatic activities of MMP-2 and MMP-9 gelatinases were measured following the method previously described (Stan et al., 2015). Briefly, the samples corresponding to 50 μg of protein (prepared in non-reducing conditions and not thermally denaturated prior to their loading on gel) were separated on 7.5% SDS-PAGE containing 0.2% gelatin. Further, the gels were washed two times with distilled water and incubated two times for 15 min with renaturing buffer (50 mM Tris HCl, pH 7.6, 2.5% Triton X-100), again washed with water and incubated overnight at 37°C under mild shacking with activation buffer (50 mM Tris HCl, pH 7.6, 10 mM CaCl_2_, 50 mM NaCl, 0.05% Brij 35). Next, gels were stained with Coomassie Brilliant Blue G-250, destained with 20% ethanol – 10% acetic acid and imaged at the Chemidoc MP system. The white bands on the blue gel, corresponding to MMPs activity, were quantified with GELQUANT.NET software.

### Histopathology

Tissue sections were fixed in 10% neutral buffered formalin and embedded in paraffin. Paraffin sections were stained with hematoxylin–eosin (H&E) or Fouchet van Gieson according to the protocol provided with the Bio-Optica staining kits. Sections were examined using an Olympus BX43 microscope and photographed using a digital camera (Olympus XC30).

Fibrosis was graded and adapted to the method of Gui et al. (2006): grade 0 – normal; grade 1 – fibrosis present (collagen fiber present as small septa); grade 2 – mild fibrosis (collagen fiber extended as septa from portal tract to central vein forming incomplete septa); grade 3 – moderate fibrosis (collagen fibers formed thin complete septa); and grade 4 – severe fibrosis (collagen fibers formed thick septa).

Each sample was observed at 20× magnification. The degree of liver damage was expressed as the mean of 10 fields of view on each slide.

### Immunohistochemistry

The primary antibodies used were rabbit polyclonal TGF-β1, rabbit polyclonal Smad 2/3, mouse monoclonal α-SMA, rabbit polyclonal Anti-Col-I, rabbit polyclonal NF-κB p65 and mouse monoclonal Anti-MMP-1 antibodies. Briefly, the sections from representative paraffin-embedding tissue samples were deparaffinized, rehydrated, and incubated with the primary antibody (1:100 dilution) overnight at 4°C. Detection was performed using a polymer detection system (Novolink max Polymer detection system, Novocastra Leica Biosystems) and DAB (3,30-diaminobenzidine, Novocastra Leica Biosystems) as chromogenic substrate. Hematoxylin staining was applied before dehydration and mounting. Negative controls included substitution of the first antibody with normal rabbit or mouse serum. A section of the model group was used as positive control. Images were acquired by light microscopy (Olympus BX43, Hamburg, Germany).

The NF-κB p65 cytoplamatic and nuclear staining was assessed on ten separate high-power fields (200×) were randomly chosen for each section, and five mice of each group were examined. The percentage of hepatocytes with only nuclear NF-κB p65 staining out of the total number of hepatocytes in each group taken at 200× magnification was calculated.

### Transmission Electron Microscopy (TEM)

Liver samples were prefixed in 2.7% glutaraldehyde solution in 0.1 M phosphate buffer, washed in 0.15 M phosphate buffer (pH 7.2), post-fixed in 2% osmic acid solution in 0.15 M phosphate buffer, dehydrated in acetone and then embedded in the epoxy embedding resin Epon 812. Ultrathin sections were double contrasted with solutions of uranyl acetate and lead citrate and were analyzed with TEM Tecnai 12 Biotwin electron microscope.

### Quantitative Real-Time PCR Analysis

Gene expression of Col I, TIMP-1, MMP-1, MMP-2, and MMP-9 were determined using real-time quantitative polymerase chain reaction (qPCR).

Liver samples were collected from the mice in RNA later solution (Thermo Scientific). Total RNA was isolated using SV Total RNA Isolation kit (Promega) according to the manufacturer’s protocol. The quantity and quality of purified RNA was assessed using a NanoDrop 8000 spectrophotometer (Thermo Scientific), and was afterwards reverse transcribed to corresponding cDNA, using First Strand cDNA Synthesis Kit (Thermo Scientific). Conditions for the reverse transcriptase reaction were: 25°C for 5 min, 37°C for 60 min and 70°C for 5 min. Real-time PCR was performed using Maxima SYBR Green/ROX qPCR Master Mix (Life Technologies) with Mx3000P^TM^ real-time PCR system. All samples were run in triplicate.

The primers used for NF-κB 50, NF-κB 65, TNF-α, IL-6, TGF-β1, α-SMA, Smad-2, -3, and -7, Col I, TIMP-1, MMP-1, -2, -9 mRNA detection are presented in **Table 1**. The mRNA levels of target genes were normalized to the levels of glyceraldehyde 3-phosphate dehydrogenase (GAPDH), which was used as reference gene and was assessed under the same experimental protocol. Relative expression changes were determined using the 2ΔΔ*C*(T) method (Livak and Schmittgen, 2001).

**Table 1:** Primer sequences for RT-PCR.

Target	Sense	Antisense
NF-κB 50	5′-AGGAAGAAAATGGCGGAGTT-3′	5′-GCATAAGCTTCTGGCGTTTC-3′
NF-κB 65	5′-CTTGGCAACAGCACAGACC-3′	5′-GAGAAGTCCATGTCCGCAAT-3′
TNF-α	5′CTGTAGCCCACGTCGTAGC3′	5′ TTGAGATCCATGCCGTTG 3′
IL-6	5′AAA GAG TTG TGC AAT GGC AAT TCT 3′	5′ AAG TGC ATC ATC GTT GTT CAT ACA 3′
TGF-β1	5′-TTTGGAGCCTGGACACACAGTACA-3′	5′-TGTGTTGGTTGTAGAGGGCAAGGA-3′
α-SMA	5′-CCGACCGAATGCAGAAG GA-3′	5′-ACAGAGTATTTGCGCTCCGAA-3′
Smad 2	5′-GTTCCTGCCTTTGCTGAGAC-3′	5′-TCTCTTTGCCAGGAATGCTT-3′
Smad 3	5′-TGCTGGTGACTGGATAGCAG-3′	5′-CTCCTTGGAAGGTGCTGAAG-3′
Smad 7	5′-GCTCACGCACTCGGTGCTCA-3′	5′-CCAGGCTCCAGAAGAAGTTG-3′
Col I	5′CAGCCGCTTCACCTACAGC 3′	5′ TTTTGTATTCAATCACTGTCTTGCC 3′
TIMP-1	5′GGTGTGCACAGTGTTTCCCTGTTT 3′	5′TCCGTCCACAAACAGTGAGTGTCA 3′
MMP-1	5′-GCAGCGTCAAGTTTAACTGGAA-3′	5′-AACTACATTTAGGGGAGAGGTGT-3′
MMP-2	5′CAG GGA ATG AGT ACT GGG TCT ATT 3′	5′ ACT CCA GTT AAA GGC AGC ATC TAC 3′
MMP-9	5′GGACCCGAAGCGGACATTG 3′	5′ CGTCGTCGAAATGGGCATCT 3′
GAPDH	5′-CGACTTCAACAGCAACTCCCACTCTTCC-3′	5′-TGGGTGGTCCAGGGTTTCTTACTCCTT-3′

### Statistical Analysis

Data were statistically processed using GraphPad Prism 3.03 software (GraphPad Software, Inc., La Jolla, CA, United States), and one-way analysis of variance, followed by a Bonferroni test. *p* < 0.05 was considered to indicate a statistically significant difference.

## Results

### Phase-Solubility Analysis

**FIGURE 2:**
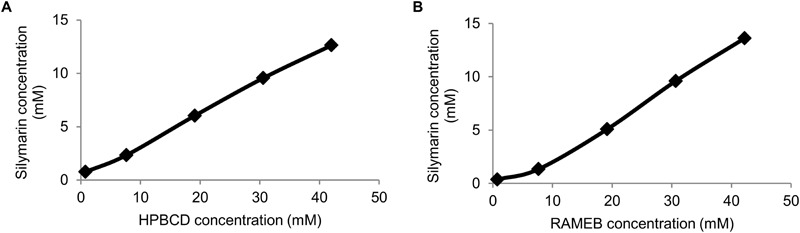
Phase-solubility diagrams of Sy-HPBCD **(A)** and Sy-RAMEB **(B)** complexes.

Phase-solubility profiles were determined in order to characterize the effect of cyclodextrins on silymarin solubility. **Figure 2** shows the solubility profiles of silymarin in the presence of HPBCD and RAMEB. Both cyclodextrin derivatives were able to improve the water solubility of silymarin in a cyclodextrin concentration dependent manner. Solubility enhancement was calculated using *S*_0_ and the highest silymarin concentration solubilized. According to this parameter, RAMEB and HPBCD have similar abilities to improve silymarin solubility in water. Contrary, sylimarin – cyclodextrin physical mixture has been shown to have much lower solubility than complexes (**Supplementary Figures S1, S2**).

Randomly methylated complexes had slightly higher stability constant value with silymarin than HPBCD, and their complexation efficiency (CE) values showed a similar relationship (**Table 2**). CE values demonstrated that in solution, only a small proportion of cyclodextrin molecules forms water soluble complexes with silymarin. It means that only about one out of three cyclodextrin molecules form a silymarin complex. As a consequence, we used an excess amount of cyclodextrins in our further experiments in order to guarantee the perfect dissolution of silymarin in water and to avoid precipitation under dilution.

**Table 2:** Complexation efficiency (CE), stability constant of 1:1 complex of silymarin formed with HPBCD and RAMEB (*K*_1:1_) and improvement of dissolution calculated from the phase-solubility curves of silymarin complexed with HPBCD and RAMEB.

	CE	*K*_1:1_	*S*_0_ (mM)	Sy solubility enhancement
HPBCD	0.42	890	0.467	27
RAMEB	0.49	1060	0.467	29

### SEM Analysis

**FIGURE 3:**
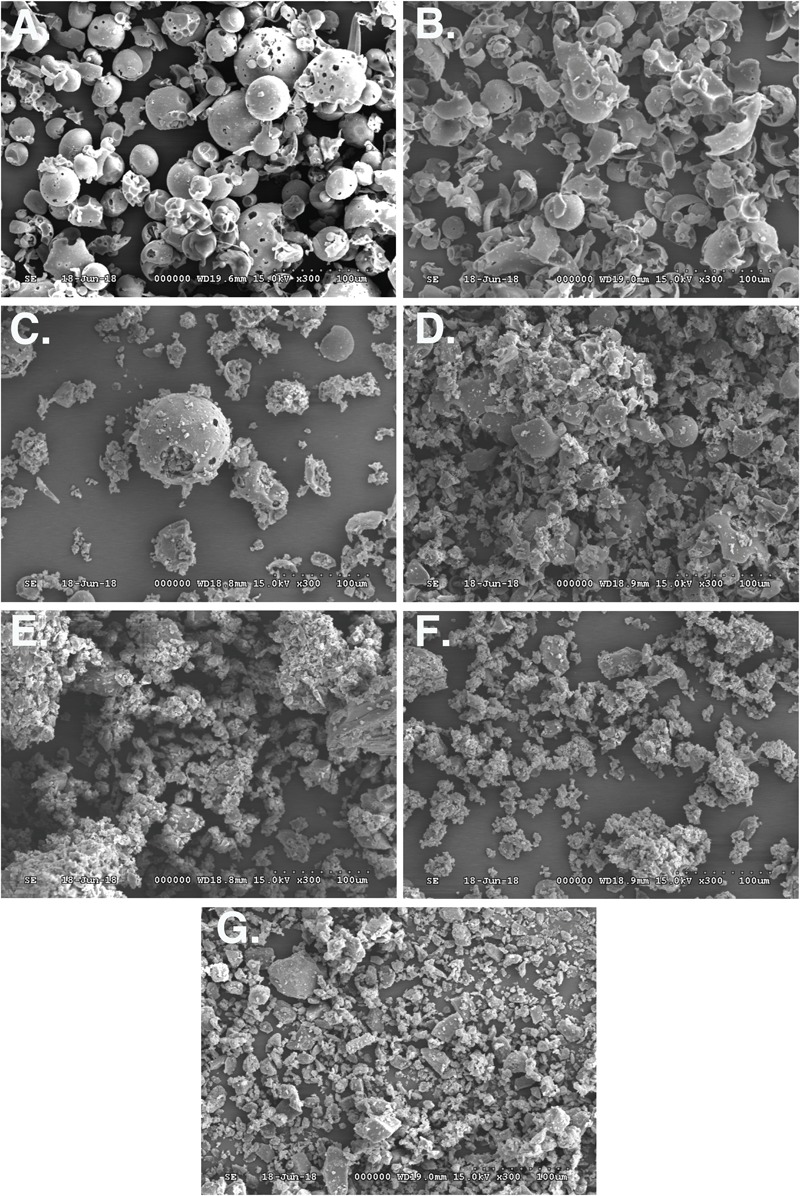
Scanning electron microscopy (SEM) images of HPBCD **(A)**, RAMEB **(B)**, silymarin-HPBCD physical mixture **(C)**, silymarin-RAMEB physical mixture **(D)**, silymarin-HPBCD complex **(E)**, silymarin-RAMEB complex **(F)**, free silymarin **(G)**.

The morphology of raw silymarin, silymarin-cyclodextrin complexes and silymarin-cyclodextrin physical mixtures were examined by SEM (**Figure 3**). Raw silymarin showed various block shape with wide particle size distribution. HPBCD particles exhibited spherical shape (**Figure 3A**), while images of RAMEB (**Figure 3B**) showed, that most of the spherical particles were broken. The images of physical mixtures clearly show, that the small particles of silymarin are attached on the surface of cyclodextrin particles and together form aggregates (**Figures 3C,D**). Silymarin-HPBCD or RAMEB complexes show totally different morphology (**Figures 3E,F**). After the complexation process and grinding the original cyclodextrin and silymarin particles cannot be identified, but aggregates containing smaller particles reveal the interaction between silymarin and cyclodextrins. The formed new structure contributes to the higher solubility and improved bioavailability of silymarin.

### Sy-HPBCD and Sy-RAMEB Complexes Reduce Oxidative Damage and Increase Antioxidant Enzymes Activities

The oxidative stress induced by CCl_4_ in the liver (**Table 3**) was shown by the increased levels of MDA, carbonyl groups and AOPP, which were, respectively, 2.7, 1.4, and 2.1 times, significantly higher compared to control group. In addition, a reduction by 50% in GSH content confirmed the CCl_4_-induced toxicity in hepatic cells. A period of 2 weeks left after the CCl_4_ administration as a spontaneous reversion of hepatic damage did not succeed to shift the values of MDA, GSH, and AOPP near to those of control group, although the levels were not as significantly changed as for the CCl_4_-treated mice. Only the content of carbonyl groups was almost similar with that of control. Regarding the benefic effect of silymarin against the hepatic toxicity induced by CCl_4_, the Sy-HPBCD formulation showed the best ability to inhibit the negative action of CCl_4_, being the protein contents, the lipid oxidation products and GSH levels similar to control mice. In contrast, the other two forms of silymarin were not as effective as Sy-HPBCD in alleviating the hepatic oxidative stress: an increase with 60 and 10% of MDA level and a decrease by 25 and 14% of GSH concentration compared to control were observed for CCl_4_/Sy and CCl_4_/Sy-RAMEB groups, respectively.

**Table 3:** The values of MDA, GSH, carbonyl groups, and AOPP concentrations after the treatment with CCl_4_ and various forms of silymarin.

Animal group	Control	CCl_4_	CCl_4_ control	CCl_4_/Sy-RAMEB	CCl_4_/Sy-HPBCD	CCl_4_/Sy
MDA (nmoles/mg protein)	0.036 ± 0.012	0.099 ± 0.022 ^∗∗∗^	0.065 ± 0.025^#^	0.039 ± 0.021^###^	0.036 ± 0.014^###^	0.057 ± 0.015^##^
GSH (nmoles/mg protein)	1.59 ± 0.46	0.81 ± 0.25 ^∗∗^	1.13 ± 0.19^##^	1.37 ± 0.34^##^	1.67 ± 0.49^#^	1.20 ± 0.26^#^
Carbonyl groups (nmoles/mg protein)	9.55 ± 0.59	13.10 ± 0.90 ^∗∗∗^	9.70 ± 1.68^###^	9.4 ± 0.99^###^	9.46 ± 1.83^###^	10.33 ± 0.72^###^
AOPP (nmoles/mg protein)	55.88 ± 34.3	119.1 ± 25.0 ^∗∗^	76.80 ± 57.8^#^	61.42 ± 39.3^##^	57.54 ± 26.6^###^	64.91 ± 19^##^

### Sy-HPBCD and Sy-RAMEB Complexes Alleviate CCl_4_-Induced Structural Changes in Liver

**FIGURE 4:**
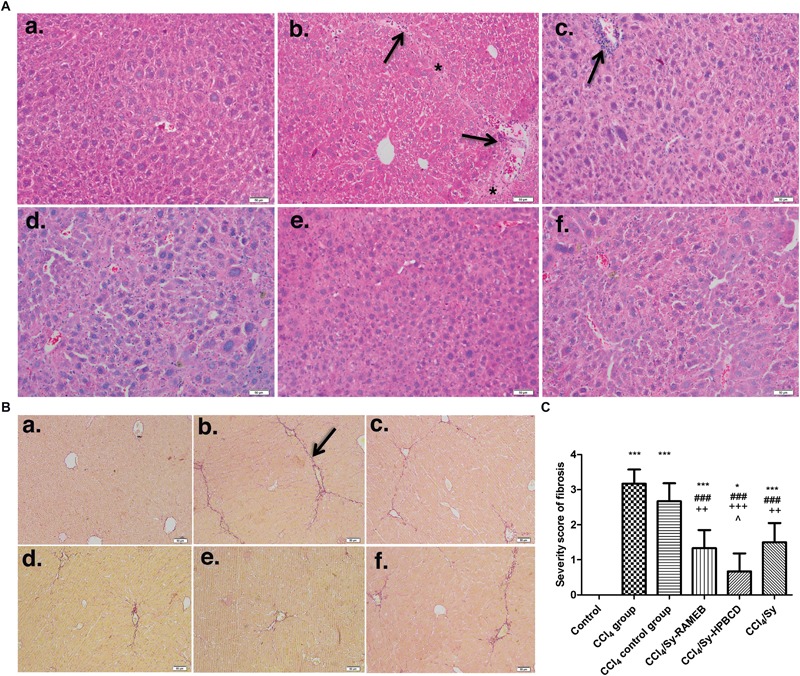
Effect induced by Sy-HPBCD and Sy-RAMEB complexes on the histological changes in liver of CCl_4_-treated mice. **(A)** H&E. **(a)** Control group: normal lobular architecture and cell structure; **(b)** CCl_4_-treated group: extensive hepatocellular damage with the presence of inflammatory cell infiltration, steatosis and necrosis, pseudo-lobular formation with collagen deposition; **(c)** CCl_4_-control group: the recovery was still weak and aspect almost similar to CCl_4_-treated group; **(d)** CCl_4_ and Sy-RAMEB co-treated group: mild inflammation, minimal hepatocellular necrosis and less extended fibrotic septa; **(e)** CCl_4_ and Sy-HPBCD co-treated group: histological aspect closest to the control with minimal inflammatory cells; **(f)** CCl_4_ and free Sy co-treated group: minimal fibrotic changes are present. ^∗^Fibrotic septa and collagen deposition; arrow – inflammatory cell infiltration; scale bar – 50 μm. **(B)** Fouchet van Gieson trichrome. **(a)** Control group: no significant collagen deposition; **(b)** CCl_4_-treated group: substantial collagen deposition in the periportal and pericellular areas, large fibrous septa formation, pseudo-lobe separation; **(c)** CCl_4_-control group: the recovery was still weak and aspect almost similar with CCl_4_-treated group; **(d)** CCl_4_ and Sy-RAMEB co-treated group: less collagen deposition was observed than that in mice treated with CCl_4_ alone; **(e)** CCl_4_ and Sy-HPBCD co-treated group: histological aspect closest to the control; **(f)** CCl_4_ and free Sy co-treated group: fibrotic changes are still present; arrow- fibrotic septa and collagen deposition; scale bar – 50 μm. **(C)** Histogram showing the percentage area of Fouchet van Gieson staining of collagen. ^∗^*p* < 0.05 compared to control; ^∗∗∗^*p* < 0.001 compared to control; ^###^*p* < 0.001 compared to CCl4 group; ^++^*p* < 0.01 compared to CCl4 control group; ^+++^*p* < 0.001 compared to CCl4 control group; ˆ*p* < 0.05 compared to CCl4/Sy group.

Liver fibrosis was evaluated in mice by two histological methods, H&E and Fouchet van Gieson’s trichrome stain, both assays showing the same pattern (**Figure 4**). The histological analysis of the livers harvested from control mice indicated a normal liver lobular architecture with central vein and radiating hepatic cords, without any proliferation of connective tissue (**Figure 4A-a**). Liver specimens from CCl_4_ group showed severe changes in morphology, including necrosis, obvious collagen deposition, formation of pseudo-lobules, and infiltration of inflammatory cells in liver interstitial areas. Hepatocytes with macro- or micro-vesicular steatosis and congestion of sinusoids were also observed (**Figure 4A-b**). However, an important degree of liver fibrogenesis and formation of pseudo-lobules, accompanied with inflammatory cell infiltration were still pronounced after 2 weeks of recovery (CCl_4_ control group), compared with CCl_4_ group. The lesions in free silymarin-treated mice were present to a lesser degree than those found in the CCl_4_-treated group, but significantly small hepatic fibrosis areas and inflammatory infiltrations in the periportal areas were still evident. In mice treated with Sy-HPBCD or Sy-RAMEB, the livers showed maintained histo-architecture, almost similar to control (**Figures 4A-d,e**).

The livers from control mice stained with Fouchet van Gieson’s trichrome showed traces of collagen only in the vascular walls (**Figure 4B-a**). As expected, repeated administration of CCl_4_ for 7 weeks caused overt bridging fibrosis in the liver (**Figure 4B-b**). Liver sections from the CCl_4_ group were characterized by tissue architecture disruption, fibers extension, large fibrous septa formation, pseudo-lobe separation, and fibers accumulation. The extent of fibrotic changes was even still pronounced in the CCl_4_ control group (*p* < 0.001) compared to control (**Figure 4B-c**). Treatment with Sy-HPBCD or Sy-RAMEB has significantly reduced the score of liver fibrosis, compared to CCl_4_ group (*p* < 0.001). The degree of liver fibrosis and thickness of fibrous septa were significantly decreased in Sy-HPBCD group compared to Sy-RAMEB one (*p* < 0.05) (**Figures 4B-d,e**), whereas free silymarin group exhibited almost similar fibrosis score with that treated with RAMEB inclusion complexes (**Figure 4B-f**).

### Sy-HPBCD and Sy-RAMEB Complexes Down-Regulates NF-κB Signaling and Inflammatory Cytokines

**FIGURE 5:**
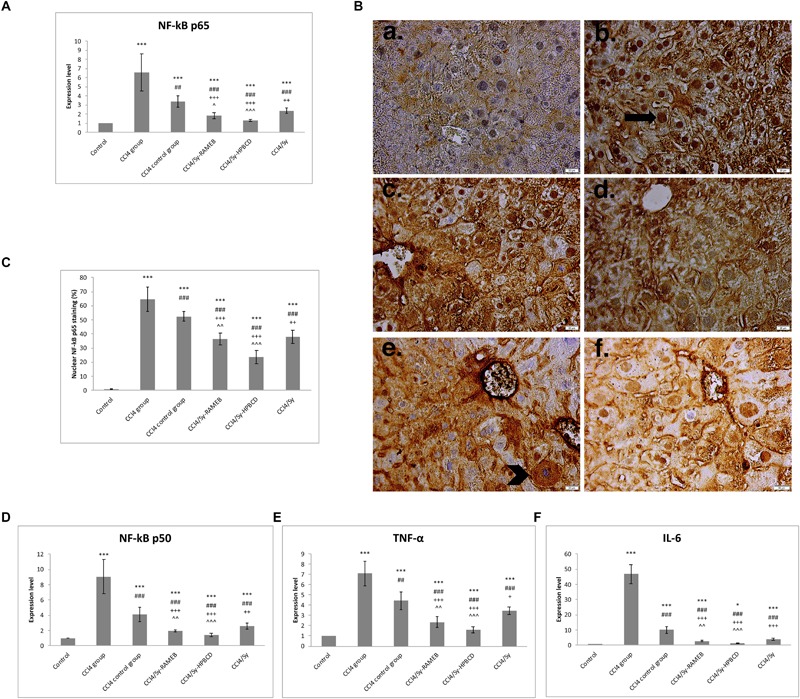
Effects of Sy-HPBCD and Sy-RAMEB complexes on nuclear translocation of NF-kB in hepatocytes and inflammatory cytokines down-regulation in liver fibrosis. mRNA expression of NF-κB p65 **(A)**, NF-κB p50 **(D)**, TNF-α **(E)** and IL-6 **(F)**; Immunohistochemical staining of NF-κB p65 **(B)**; arrow – hepatocytes with only nuclear NF-κB p50 staining; arrowhead – hepatocytes with only cytoplasmic NF-κB p50 staining. The percentage of hepatocytes with only nuclear NF-kB p65 staining out of the total number of hepatocytes was calculated **(C)**. Data are expressed as the arithmetic mean ± standard deviation (SD) of five mice per group; ^∗∗∗^*p* < 0.001 compared to control; ^###^*p* < 0.001 compared to CCl_4_ group; ^##^*p* < 0.01 compared to CCl_4_ group; ^+++^*p* < 0.001 compared to CCl_4_ control group; ^++^*p* < 0.01 compared to CCl_4_ control group; ˆˆ*p* < 0.01 compared to CCl_4_/Sy group; ˆˆˆ*p* < 0.001 compared to CCl_4_/Sy group. ^+^*p* < 0.05 compared to CCl_4_ group; ^∗^*p* < 0.05 compared to control.

Significant increase in NF-κB p50, NF-κB p65, TNF-α and IL-6 mRNA expressions were detected in CCl_4_-induced liver fibrosis in mice, compared to control (**Figures 5A,D–F**). Fourteen days of daily Sy-HPBCD or Sy-RAMEB oral administration induces significant down-regulation of all genes compared to CCl_4_ group (*p* < 0.001). The anti-inflammatory activity of Sy-HPBCD has been more highlighted.

Therefore we tracked activation of NF-κB p65 in livers by immunohistochemistry analysis (**Figure 5B**). In CCl_4_ and CCl_4_ control groups the p65 protein concentrated in the nucleus (**Figure 5C**; *p* < 0.001), whereas in silymarin-cyclodextrin treated groups p65, the expression decreased significant and localized nearly entirely to the cytosol.

### Sy-HPBCD and Sy-RAMEB Complexes Treatment Inhibits Activation and Proliferation of Hepatic Stellate Cells (HSCs)

**FIGURE 6:**
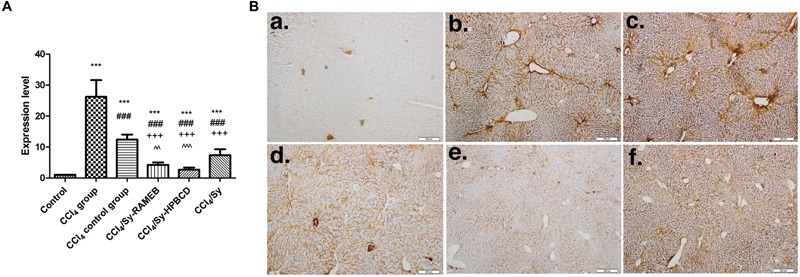
The mRNA expression and specific tissue distribution of α-SMA. **(A)** RT-PCR analysis of α-SMA gene level. ^∗∗∗^*p* < 0.001 compared to control; ^###^*p* < 0.001 compared to CCl_4_ group; ^+++^*p* < 0.001 compared to CCl_4_ control group; ˆˆ*p* < 0.01 compared to CCl_4_/Sy group; ˆˆˆ*p* < 0.001 compared with CCl_4_/Sy group. **(B)** Immunohistochemical expression of α-SMA in experimental livers. **(a)** Control group, **(b)** CCl_4_-treated group, **(c)** CCl_4_-control group, **(d)** CCl_4_ and Sy-RAMEB co-treated group, **(e)** CCl_4_ and Sy-HPBCD co-treated group, **(f)** CCl_4_ and free Sy co-treated group.

The expression of α-SMA is a characteristic feature of activated HSCs and is considered one of the important markers of hepatic fibrosis. The RT-PCR analysis showed significantly higher α-SMA gene expression level for CCl_4_ group (*p* < 0.001). Both of Sy-HPBCD and Sy-RAMEB groups presented significantly decreased levels of gene expression, by about 9.91- and 5.24-folds, lower compared to CCl_4_ group (*p* < 0.001). For both of silymarin-cyclodextrin complexes the decrease was statistically significant compared to free Sy group (**Figure 6A**).

In the control livers, α-SMA-immunopositive cells were absent. For CCl_4_ group, intensely stained α-SMA-positive HSCs associated with bridging fibrotic septa were present (**Figure 6B**). We found the same pattern for the spontaneous reversal of fibrosis group (CCl_4_ control group). However, α-SMA-positive cells could be found nearness of fibrotic lesions in free Sy group, while mice treated with Sy-HPBCD or Sy-RAMEB had staining pattern almost similar to control animals.

### Sy-HPBCD and Sy-RAMEB Complexes Down-Regulate TGF-β1/Smad Signaling Pathway

Transforming growth factor-β1 has been long considered a key mediator in the pathogenesis of liver fibrosis, being a major pro-fibrotic cytokine that leads to the activation of HSCs to secrete compounds of ECM (Novo et al., 2014). Therefore, we examined if Sy-HPBCD and Sy-RAMEB are able to inhibit the TGF-β1 expression in mice with CCl_4_-induced hepatotoxicity.

**FIGURE 7:**
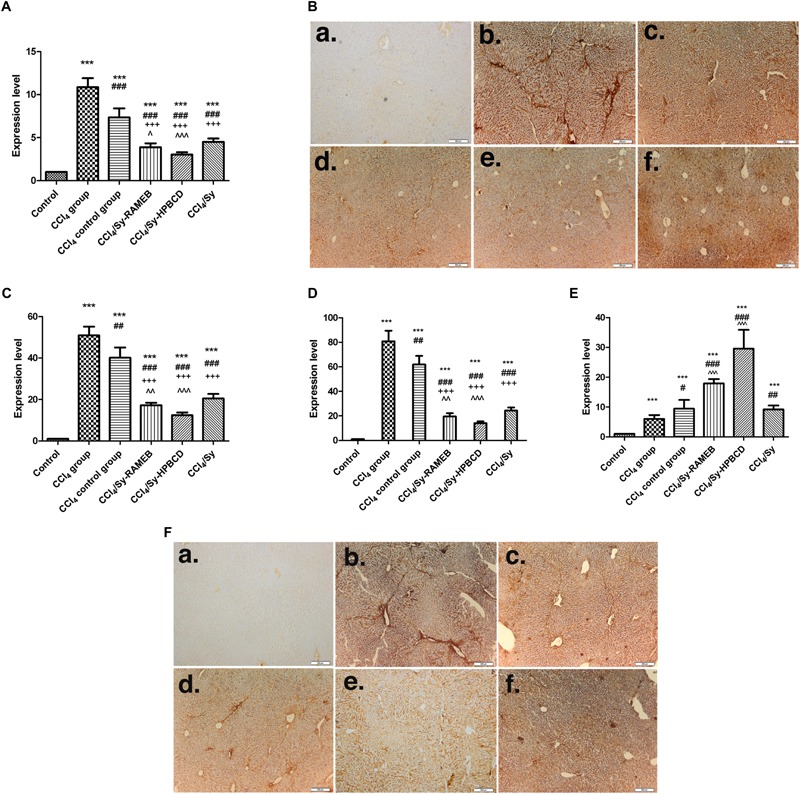
Effects of Sy-HPBCD and Sy-RAMEB complexes on TGF-β1/Smad signaling pathway. **(A)** RT-PCR analysis of TGF-β1 gene level. ^∗∗∗^*p* < 0.001 compared to control; ^###^*p* < 0.001 compared to CCl_4_ group; ^+++^p < 0.001 compared to CCl_4_ control group; ˆ*p* < 0.05 compared to CCl_4_/Sy group; ˆˆˆ*p* < 0.001 compared to CCl_4_/Sy group. **(B)** Immunohistochemical expression of TGF-β1 in experimental livers. **(a)** Control group, **(b)** CCl_4_-treated group, **(c)** CCl_4_-control group, **(d)** CCl_4_ and Sy-RAMEB co-treated group, **(e)** CCl_4_ and Sy-HPBCD co-treated group, **(f)** CCl_4_ and free Sy co-treated group; RT-PCR analysis of Smad 2 **(C)**, Smad 3 **(D),** and Smad 7 **(E)** gene levels. ^∗∗∗^*p* < 0.001 compared to control; ^#^*p* < 0.05 compared to CCl_4_ group; ^##^*p* < 0.01 compared to CCl_4_ group; ^###^*p* < 0.001 compared to CCl_4_ group; ^+++^*p* < 0.001 compared to CCl_4_ control group; ˆˆ*p* < 0.01 compared to CCl_4_/Sy group; ˆˆˆ*p* < 0.001 compared to CCl_4_/Sy group. **(F)** Immunohistochemical expression of Smad 2/3 in experimental livers. **(a)** Control group, **(b)** CCl_4_-treated group, **(c)** CCl_4_-control group, **(d)** CCl_4_ and Sy-RAMEB co-treated group, **(e)** CCl_4_ and Sy-HPBCD co-treated group, **(f)** CCl_4_ and free Sy co-treated group.

CCl_4_-induced liver fibrosis was associated with an important up-regulation of TGF-β1 gene expression (*p* < 0.001). Compared to CCl_4_ group, the TGF-β1 mRNA levels for Sy-HPBCD or Sy-RAMEB groups were significantly reduced by about 72.02, and 64.2%, respectively, whereas in the CCl_4_ control group its levels were found significantly higher compared to all flavonoid co-treated groups (*p* < 0.001) (**Figure 7A**). The protective response to free Sy co-treatment was lower by about 0.48-fold, respectively, 0.15-fold compared to Sy-HPBCD and Sy-RAMEB groups.

Immunohistochemical expression of TGF-β1 in the control group was not detectable (**Figure 7B-a**). The CCl_4_ administration significantly induced TGF-β1 immunoreactivity within the fibrotic septa in non-parenchymal cells (**Figure 7B-b**). Similarly, Kupffer cells strongly expressing TGF-β1 were infiltrated in necrotic area around the central vein and fibrotic septa in samples of CCl_4_ control group. Free silymarin treatment significantly reduced hepatic TGF-β1 expression (**Figure 7B-f**), which was completely withdrawn by 50 mg/kg Sy-HPBCD or 50 mg/kg Sy-RAMEB administration for 2 weeks (**Figures 7B-d,e**).

The signaling from TGF-β1 receptors to the nucleus occurs by phosphorylation of several cytoplasmic proteins belonging to the Smad family (Verrecchia and Mauviel, 2002). TGF-β1 is known to exert its fibrogenic effect through phosphorylation of Smad 2/3. Furthermore, Smad 7 is an inhibitory Smad that negatively regulates Smad 2/3 activation and functions by targeting the TGF-β1 receptor (Zhang et al., 2014).

As shown in **Figures 7C–E**, Smad 2, -3, and -7 mRNA levels were significantly up-regulated after 7 weeks of CCl_4_ administration (*p* < 0.001). Two weeks of Sy-HPBCD or Sy-RAMEB oral administration led to a significant decrease of Smad 2 mRNA levels, by 75.7%, respectively, 66.21%, whereas Smad 3 mRNA expression down-regulated by 82.65 and 75.91%, respectively, compared with CCl_4_ group. Otherwise, our results showed significant up-regulation of Smad 7 mRNA expression, by 79.71 and 66.49% for Sy-HPBCD and Sy-RAMEB, respectively, in comparison with CCl_4_ group. Both Smad 2 and Smad 3 gene expressions were significantly down-regulated in Sy-HPBCD group compared to free Sy group (*p* < 0.001). Also, the Smad 7 one was significantly up-regulated in both Sy-HPBCD and Sy-RAMEB groups compared to free Sy group (*p* < 0.001). After 2 weeks of *de novo* recovery (CCl_4_ control group), the levels of Smad 2, -3 and -7 proteins were lower, compared to all three flavonoid co-treated groups. Immunohistochemical expression of Smad 2/3 showed the same pattern (**Figure 7F**).

### Sy-HPBCD and Sy-RAMEB Complexes Down-Regulate Col 1 and Decrease Deposition of Collagen in Hepatic Tissue

**FIGURE 8:**
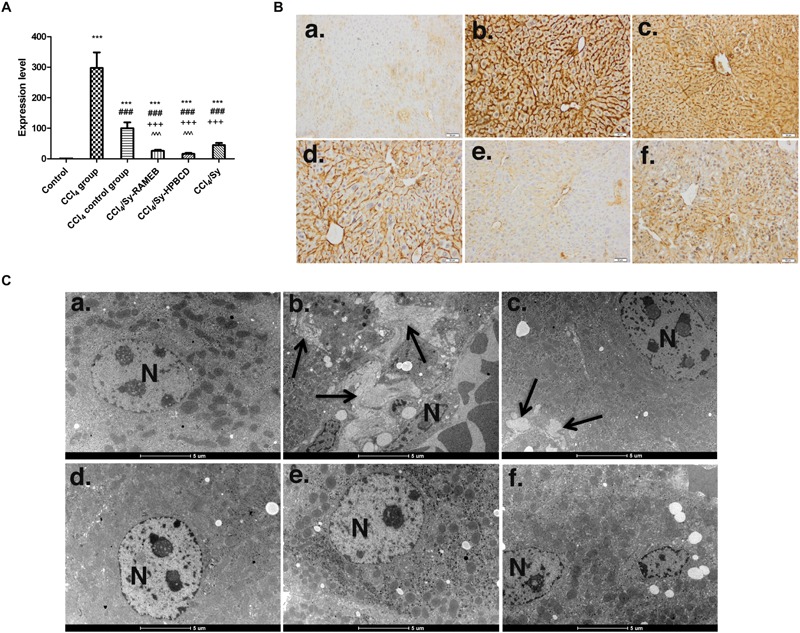
Effects of Sy-HPBCD and Sy-RAMEB complexes on collagen over-production. **(A)** RT-PCR analysis of Col 1 gene level. ^∗∗∗^*p* < 0.001 compared to control; ^###^*p* < 0.001 compared to CCl_4_ group; ^+++^*p* < 0.001 compared to CCl_4_ control group; ˆ*p* < 0.05 compared to CCl_4_/Sy group; ˆˆˆ*p* < 0.001 compared to CCl_4_/Sy group. **(B)** Immunohistochemical expression of Col 1 in experimental livers. **(a)** Control group, **(b)** CCl_4_-treated group, **(c)** CCl_4_-control group, **(d)** CCl_4_ and Sy-RAMEB co-treated group, **(e)** CCl_4_ and Sy-HPBCD co-treated group, **(f)** CCl_4_ and free Sy co-treated group. **(C)** Collagen deposition in experimental livers by electron microscopy. **(a)** Control group, **(b)** CCl_4_-treated group, **(c)** CCl_4_-control group, **(d)** CCl_4_ and Sy-RAMEB co-treated group, **(e)** CCl_4_ and Sy-HPBCD co-treated group, **(f)** CCl_4_ and free Sy co-treated group; Collagen fibers (arrow); N, nucleus.

CCl_4_-induced liver fibrosis was associated with a marked up-regulation of Col 1 gene expression (*p* < 0.001). Compared with CCl_4_ group, the Col 1 mRNA levels for Sy-HPBCD or Sy-RAMEB groups were significantly reduced by about 6.26%, respectively, 3.89% compared to control, whereas in the CCl_4_ control group its level was found significantly higher compared to all flavonoid co-treated groups (*p* < 0.001) (**Figure 8A**). The protective response to free Sy co-treatment was lower by about 2.77-fold, respectively, 1.72-fold compared to Sy-HPBCD and Sy-RAMEB groups (**Figure 8A**).

Immunohistochemical expression of Col-1 follows the same pattern (**Figure 8B**).

The collagen deposition was confirmed by electron microscopy, as shown in **Figure 8C**. Electron microscopy micrographs of fibrotic group (**Figure 8C-b**) highlights dense bundle of collagen fibers proliferates in the parenchima, space of Disse and between swollen profiles of a sinusoid endothelial cell, and maintained in self recovery group (**Figure 8C-c**). The ultrastructure of livers were alleviates on Sy-HPBCD, Sy-RAMEB and Sy-treated livers (**Figures 8C-d–f**).

### Sy-HPBCD and Sy-RAMEB Complexes Modulate ECM by TIMP-1/MMPs Balance

**FIGURE 9:**
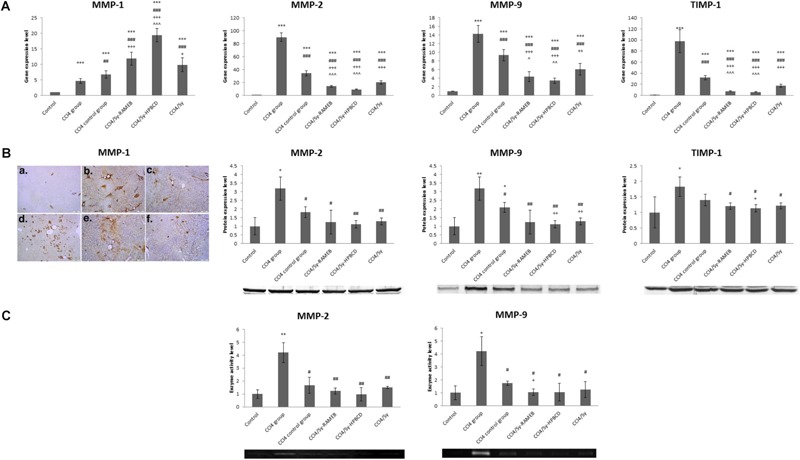
Effects of Sy-HPBCD and Sy-RAMEB complexes on TIMP-1/MMPs modulation. **(A)** RT-PCR analysis of MMP-1, MMP-2, MMP-9, and TIMP-1 mRNA expression levels. **(B)** Immunohistochemical expression of MMP-1 and Western Blot analysis of MMP-2, MMP-9, and TIMP-1 protein expression levels. **(C)** Zymography analysis of MMP-2 and MMP-9 enzyme activity levels. ^∗^*p* < 0.05, ^∗∗^*p* < 0.01, ^∗∗∗^*p* < 0.001 compared to control; ^#^*p* < 0.05, ^##^*p* < 0.01, ^###^*p* < 0.001 compared to CCl_4_ group; ^+^*p* < 0.05, ^++^*p* < 0.01, ^+++^*p* < 0.001 compared to CCl_4_ control group; ˆ*p* < 0.05, ˆˆ*p* < 0.01 compared to CCl_4_/Sy group. ˆˆˆ*p* < 0.01 compared to CCl_4_/Sy group.

TIMP-1 is an endogenous inhibitor of matrix metalloproteinase (MMP) degradation of ECM. To investigate the inhibitory effects of Sy-HPBCD and Sy-RAMEB on ECM in the livers of mice with CCl_4_-induced hepatotoxicity, the mRNA levels of TIMP-1 and MMP-1, 2, and 9 were measured by RT-PCR analysis (**Figure 9A**). Our data showed that the expression levels of these genes were significantly increased (*p* < 0.001) in CCl_4_ group compared to control. The treatment with Sy-HPBCD or Sy-RAMEB significantly succeeded to down-regulate the mRNA levels of MMP-2, MMP-9, and TIMP-1 compared to those obtained for CCl_4_ and CCl_4_ control groups (*p* < 0.001). By contrast, the mRNA level of MMP-1 was significantly higher for Sy-HPBCD and Sy-RAMEB treated groups compared to CCl_4_ and CCl_4_ control groups (*p* < 0.001). In Sy-HPBCD group, the mRNA expressions of TIMP-1, MMP-2, and MMP-9 were lower by about 2.9-, 0.46-, and 1.77-fold, respectively, compared to free silymarin group; while gene expression of MMP-1 was significantly up-regulated compared to the flavonoid silymarin group (*p* < 0.001). Similar, for Sy-RAMEB group, the decreases of TIMP-1, MMP-2, and MMP-9 mRNA expressions were by about 2.23-, 0.7-, and 1.39-fold, respectively, compared to free silymarin group. Regarding the expression of these proteins, the immunohistochemical analysis of MMP-1 and Western Blots of MMP-2, MMP-9, and TIMP-1 revealed the same pattern as obtained for mRNA expression for each of them (**Figure 9B**). The treatment with Sy-HPBCD or Sy-RAMEB was able to diminish the protein levels of MMP-2 and MMP-9 recorded after the administration of CCl_4_. In addition, the activity of these two enzymes was elevated by 4.2 times in the livers of CCl4-treated mice and further decreased near to the control levels after the administration of Sy-HPBCD and Sy-RAMEB (**Figure 9C**).

## Discussion

Silymarin, a flavonolignan complex extracted from the seeds of *S. marianum* (milk thistle), has been traditionally used from ancient times because of its excellent hepatoprotective activity against to a large variety of liver disorders, including acute and chronic viral hepatitis, toxin/drug-induced hepatitis, alcoholic liver diseases and cirrhosis (Kshirsagar et al., 2009). The preclinical results showed that anti-fibrotic properties are based on HSC cell cycle arrest (Ezhilarasan et al., 2017), apoptosis of the activated HSCs (Tsai et al., 2008), or free radicals scavenging properties (Tzeng et al., 2012), leading to enhanced parenchymal regeneration. Due to a very poor oral bioavailability, and water solubility, silymarin is administrated in a high dose to achieve therapeutic plasma levels. Particularly, Clichici et al. (2016) demonstrated that a significant protection for liver fibrosis progression fibrosis development into the liver could be achieved by using at least 200 mg/kg Sylimarin. However, concerns about the administration of high doses of silymarin remained, while some results showed that it has a toxic dose of >1.44 g per week (Wu et al., 2011). Therefore, a number of approaches have been used for the development of novel drug delivery system in order to increase its solubility and thereby its bioavailability (Hermenean et al., 2016), which comprise as well β-cyclodextrin (β-CD) complexation (Arcari et al., 1992). Meanwhile, it has been shown that water solubility of the native β-CD can be increased, i.e., by performing a substitution of hydroxyl groups like hydroxypropyl-β-cyclodextrin (HPBCD), where the solubility in water raises from 18.5 mg/ml to >600 mg/ml (Loftsson et al., 2005a,b). Other lipophilic cyclodextrin derivates, as randomly methylated β-cyclodextrin (RAMEB), have shown better aqueous solubility (>500 mg/ml) than native β-CD (Loftsson et al., 2005a,b). In this respect therefore, we aimed to develop silymarin/β-cyclodextrin derivates complexes (Sy-HPBCD/RAMEB) with increased solubility and suitable oral bioavailability properties, in order to maximize the anti-fibrotic effect of silymarin in fibrotic livers at a lower dose of 50 mg/kg. We have to note, that we selected for *in vivo* testing only the silymarin/cyclodextrin complexes. This was based on the physical and physicochemical characterization of the complexes and physical mixtures, these data supported the use of technologically-advanced complexes. We found that silymarin had lower solubility in the physical mixtures than in the cyclodextrin complexes and the low solubility can be limiting factor of absorption (**Supplementary Figures S1, S2**). However, for complete characterization of absorption, distribution and pharmacokinetics, *in vivo* examinations would be needed and thus the interpretation of the *in vitro* tests of physical mixtures remains limited.

The hepatic toxicity induced by CCl_4_ is characterized by the induction of oxidative stress (Hermenean et al., 2015), as it was confirmed also in our study by the modified biomarkers measured, such as decreased GSH level, increased carbonyl groups amount and more than double levels of lipid peroxidation and AOPPs (**Table 3**). Antioxidant efficiency of sylimarin and its β-cyclodextrin formulations (Sy-HPBCD and Sy-RAMEB) was proved by the diminished biomarkers’ levels compared to CCl_4_-treated group. Particularly, levels assessed in the samples from mice treated with CCl_4_/Sy-HPBCD were almost comparable with those of control group, indicating that this HPBCD hydroxypropyl β-CD-based formulation had the most potent activity to alleviate the oxidative stress triggered by CCl_4_ on livers.

Nuclear factor kappa-light-chain enhancer of activated B cells (NF-κB) is a key transcription factor involved in chronic liver disease, with a particular focus on chronic inflammation and fibrosis (Papa et al., 2009). NF-κB family consists of p50 (NF-κB1), p52 (NF-κB2), p65 (RelA), c-Rel (Rel), and RelB members, of which p65, RelB and cRel contain C-terminal transactivation domains that trigger gene transcription. NF-κB p50 become active DNA-binding proteins after cleavage of p105 precursor and activate transcription when form heterodimers with subunits that contain transactivation domains, especially p65 (Luedde and Schwabe, 2011). Our results suggest that Sy-HPBCD and Sy-RAMEB complexes exerts a crucial role in inflammation alleviation and subsequent fibrosis resolution by down-regulation of the major pro-inflammatory liver cytokines as TNF-α and IL-6 through NF-κB p50 and 65 regulatory genes inhibition.

Previous studies have demonstrated a positive correlation between HSC activation and the degree of hepatic fibrosis (Novo et al., 2014). The hepatic α-SMA expression is an important and reliable marker of activated HSC *in situ* (Jarčuška et al., 2010), which is recognized as being crucial in liver fibrogenesis. Additionally, HSCs activation is the main source of excessive ECM protein synthesis and deposition. During liver damage, HSCs become activated and *trans*-differentiate into myofibroblast-like cells, being accompanied by higher proliferation, expression of α-SMA and overproduction of ECM (Friedman, 2003). Our data revealed that α-SMA hepatic expression in fibrotic mice (CCl_4_ group) was significantly increased (**Figure 4**). This was also consistent with previous observations in rodent models of CCl_4_-induced fibrosis (Amin and Mahmoud-Ghoneim, 2009; Domitrović et al., 2009; Hamza, 2010; Balta et al., 2015). Meanwhile, treatment by Sy-HPBCD and Sy-RAMEB significantly decreased α-SMA expression level in liver of CCl_4_-treated mice (**Figure 4**). These results suggest that Sy-HPBCD and Sy-RAMEB could protect mouse liver against CCl_4_-induced damage. Furthermore, the RT-PCR analysis confirmed that α-SMA mRNA hepatic level was down-regulated by Sy-HPBCD and Sy-RAMEB administration, whereas free silymarin administration exerted a lower protection (**Figure 4A**).

Transforming growth factor-β1 (TGF-β1), the most potent pro-fibrogenic cytokine, has been suggested to be an important factor in activating and promoting the transformation of HSCs (Mallat and Lotersztajn, 2013), and an active regulator of the production, degradation, and deposition of ECM proteins (Iredale et al., 2013), through Smad-dependent and -independent signaling pathways (Zhang et al., 2014). Initially, stored as an inactivated protein, once activated, TGF-β exerts its biological and pathological activities via Smad-dependent and -independent signaling pathways (Zhang et al., 2014). Thus, the TGF-β signaling pathway has become a main effective target for the prevention and therapy of hepatic fibrosis. In the present study, both types of silymarin-cyclodextrin formulations significantly attenuated the level of TGF-β1, indicating their inhibitory activity against the proliferative activity of HSCs, which might be confirmed by less fibrosis scars, collagen down-regulation and tissue deposition compared to fibrotic livers (CCl_4_ group), even after 2 weeks of recovery (CCl_4_ control group) (**Figures 5A,B**). Moreover, CCl_4_-induced liver fibrosis was associated with an important activation of Smad 2/3 expression and a lower increase of Smad 7 one, compared to control (**Figures 5C–F**). This imbalance between Smad 2/3 and Smad 7 signaling could represent also a significant event in the pathogenesis of liver fibrosis. Also, the overexpression of Smad 7 attenuated TGF-β/Smad signaling in liver and protected against HSCs activation and fibrogenesis in liver fibrotic rodent models (Bian et al., 2014). Our results showed that both Sy-HPBCD or Sy-RAMEB treatments were able to down-regulated the expression of Smad 2/3 and reversed the inhibitory effect of CCl_4_ on Smad 7 expression. The gene expression of Smad family through Sy-cyclodextrin complexes were more significant modulated than non-complexed silymarin. Therefore, we hypothesized that the decreased fibrosis level after Sy-HPBCD and Sy-RAMEB treatment might be mediated via inhibition of TGF-β1/Smad signaling pathway.

Hepatic fibrosis is a wound healing response to liver injury and is characterized by extensive deposition of ECM proteins, such as collagen types I, III, and IV, laminin as well as hyaluronic acid, leading to a decreased capability of specific catabolism (Iredale et al., 2013). In this study, the Col I mRNA expression in hepatic tissue and collagen deposits were significantly increased in CCl_4_-treated rats, whereas Sy-HPBCD and Sy-RAMEB treatment markedly down-regulated Col I, highlights that both Sy-cyclodextrin complexes can prevent collagen accumulation caused by the chronic liver injury and alleviates the development of liver fibrosis (**Figure 6**).

In the fibrotic livers, MMPs as well as their endogenous inhibitors, tissue inhibitors of metalloproteinases (TIMPs), are highly expressed. During fibrosis resolution induced by Sy-HPBCD and Sy-RAMEB administration, the mRNA TIMP-1 expression was significantly declined, tipping the overall MMPs/TIMP balance, resulting in increased matrix degrading activity and net degradation of scar tissue (Afratis et al., 2018), compared with silymarin group, as we showed in **Figures 3, 7**.

Matrix metalloproteinases collagenases are central to the process of fibrotic tissue remodeling because they cleave the native helix of fibrillar collagens and providing the gelatin susceptible to degradation by other MMPs (Iredale et al., 2013). This study showed that both Sy-HPBCD and Sy-RAMEB were able to up-regulate mRNA and protein MMP-1 (interstitial collagenase) expression and further stimulate cleavage of the native fibrillar collagens, especially Col I, by regulating the extracellular matrix balance via TIMP-1/MMP-1 components (**Figure 7**).

Matrix metalloproteinases gelatinases, as gelatinase A (MMP-2) are expressed by Kupffer cells and inflammatory macrophages (Préaux et al., 1999), respectively, gelatinase B (MMP-9) by activated stellate cells (Han et al., 2007), contribute to ECM remodeling. They are implicated in the activation of TGF-β, that is crucial for the differentiation of quiescent HSC into collagen I producing myoblasts (Zbodakova et al., 2017). Our data showed that gene expression of MMP-2 and MMP-9 remained increased after 7 weeks of CCl_4_ administration, which is in agreement with other findings (Watanabe et al., 2001; Zhou et al., 2004; Cheung et al., 2009). We also found that Sy-complexes abrogated CCl_4_-induced MMP-2/MMP-9 up-regulation. Recent studies highlighted that inhibition of MMP-2, and MMP-9 activities or blockade of their synthesis by other bioactive compounds, such as: betaine (Bingül et al., 2016), morin (Perumal et al., 2017), isoorientin (Lin et al., 2015) or helenalin (Lin et al., 2014), might effectively prevent HSC activation and proliferation and collagen accumulation.

Furthermore, it was shown the ability of both Sy-HPBCD and Sy-RAMEB to re-establish the right protein level of MMPs and TIMP-1 after the administration of CCl_4_, in order to regulate the degradation and accumulation of ECM (**Figure 7B**). The complexation of sylimarin with β-CD clearly contributed to the attenuation of liver fibrosis induced experimentally in mice by CCl_4_, as the free silymarin administration was not so efficient. This approach used within this work, the measurement of mRNA, protein and activity level of MMP-2 and MMP-9 on a long-term treatment, together with the expression of TIMP-1, allows us to validate the complex modulation of these molecules involved in the pathogenesis of liver fibrosis by Sy-HPBCD and Sy-RAMEB, in order to counteract the CCl_4_-induced damage. These results are in agreement with the attenuation of oxidative stress (**Table 3**), as it is known that reactive oxygen species, most probably over generated by CCl_4_, can activate MMPs through the oxidation of cysteine pro-domain or modification of amino acids from catalytic domain. Previously it was proved that silymarin decreased the expression of MMP-2 and MMP-9 in human melanoma cells (Vaid et al., 2011), having beyond the protective action of hepatocytes an anticancer potential (Agarwal et al., 2006).

One of the major signs of the liver fibrogenesis involved free cholesterol accumulation in HSCs and thereby stimulated the cells to TGF β-induced activation via TLR-4 signals (Tomita et al., 2014). Probably, enhanced antifibrotic effects of the complexes can be attributed additionally to β-cyclodextrin’s which can exert metabolic effects either directly through binding lipid molecules such as cholesterol or indirectly via microbiota-dependent generation of bioactive metabolites, translated in reduced plasma total cholesterol levels and lower HDL cholesterol (Mistry et al., 2017).

Taking into account that tested formulation are able to repair the oxidative damages and to modulate the turnover of ECM throughout the MMP/TIMP rebalance. Sy-HPBCD was found to be the most potent anti-fibrotic complex, but both of these can represent valid sylimarin-containing products with increased biodisponibility for treating liver fibrosis.

## Conclusion

We have demonstrated that Sy-HPBCD and Sy-RAMEB complexes decreased extracellular matrix accumulation by inhibiting HSC activation, diminished the oxidative damage and increased antioxidant defense system. Mechanistically, this might occur via inhibition of the TGF-β1/Smad signal transduction, and MMP/TIMP rebalance, by blocking the synthesis of Col I and decreasing collagen deposition. These results suggest that the complexation of silymarin with HPBCD or RAMEB are viable options for the its oral delivery, of the flavonoid as a potential therapeutic entity candidate with applications in the treatment of liver fibrosis.

## Author Contributions

AH conceived the *in vivo* experiment. SG, CB, HH, and MR performed the experiments. FF, MV, and IB performed preparation of silymarin-cyclodextrin complexes and phase-solubility study. JV performed HPLC analyses and SGy the SEM assay. SV, MS, and RC analyzed the oxidative stress and antioxidant enzymes activities. AD, SV, and MS analyzed the data. SG and AH performed the histopathology, IHC and EM studies. SG and CB performed the quantitative Real-time PCR analysis. All authors discussed the results and commented on the manuscript. AH, SG, FF, MS, and AD wrote the manuscript.

## Conflict of Interest Statement

The authors declare that the research was conducted in the absence of any commercial or financial relationships that could be construed as a potential conflict of interest.
