# Supplementary material for: Enhancement of Silymarin Anti-fibrotic Effects by Complexation With Hydroxypropyl (HPBCD) and Randomly Methylated (RAMEB) β-Cyclodextrins in a Mouse Model of Liver Fibrosis
Source: Front Pharmacol. 2018 Aug 13;9:883. doi: 10.3389/fphar.2018.00883 (PMC6099081; doi:10.3389/fphar.2018.00883)
Supplement: Supplementary file 1 [file Presentation_1.PPTX]

## Slide 1
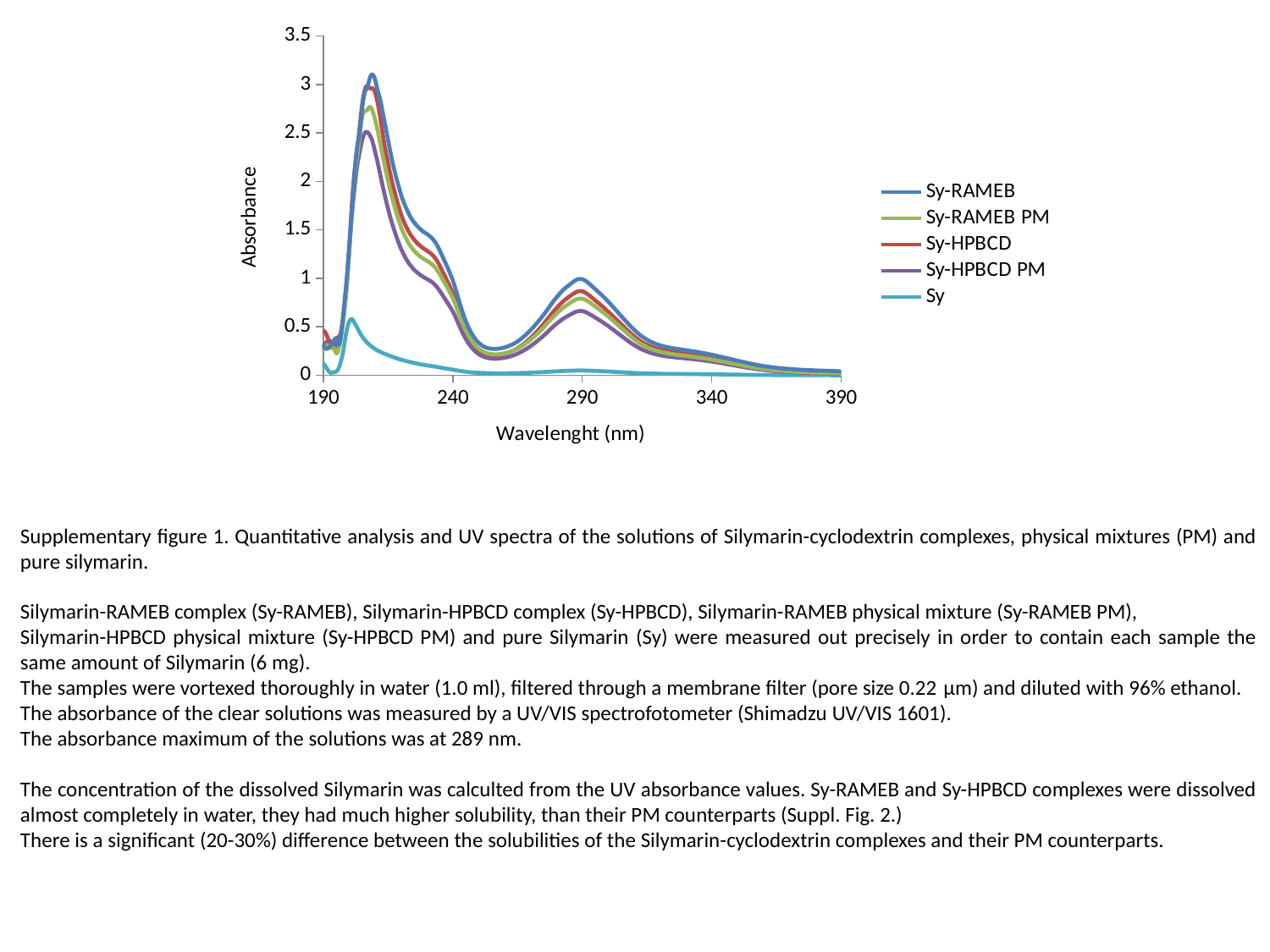

### Chart
| Category | | | | | |
|---|---|---|---|---|---|Supplementary figure 1. Quantitative analysis and UV spectra of the solutions of Silymarin-cyclodextrin complexes, physical mixtures (PM) and pure silymarin.
Silymarin-RAMEB complex (Sy-RAMEB), Silymarin-HPBCD complex (Sy-HPBCD), Silymarin-RAMEB physical mixture (Sy-RAMEB PM),
Silymarin-HPBCD physical mixture (Sy-HPBCD PM) and pure Silymarin (Sy) were measured out precisely in order to contain each sample the same amount of Silymarin (6 mg).
The samples were vortexed thoroughly in water (1.0 ml), filtered through a membrane filter (pore size 0.22 µm) and diluted with 96% ethanol.
The absorbance of the clear solutions was measured by a UV/VIS spectrofotometer (Shimadzu UV/VIS 1601).
The absorbance maximum of the solutions was at 289 nm.
The concentration of the dissolved Silymarin was calculted from the UV absorbance values. Sy-RAMEB and Sy-HPBCD complexes were dissolved almost completely in water, they had much higher solubility, than their PM counterparts (Suppl. Fig. 2.)
There is a significant (20-30%) difference between the solubilities of the Silymarin-cyclodextrin complexes and their PM counterparts.

## Slide 2
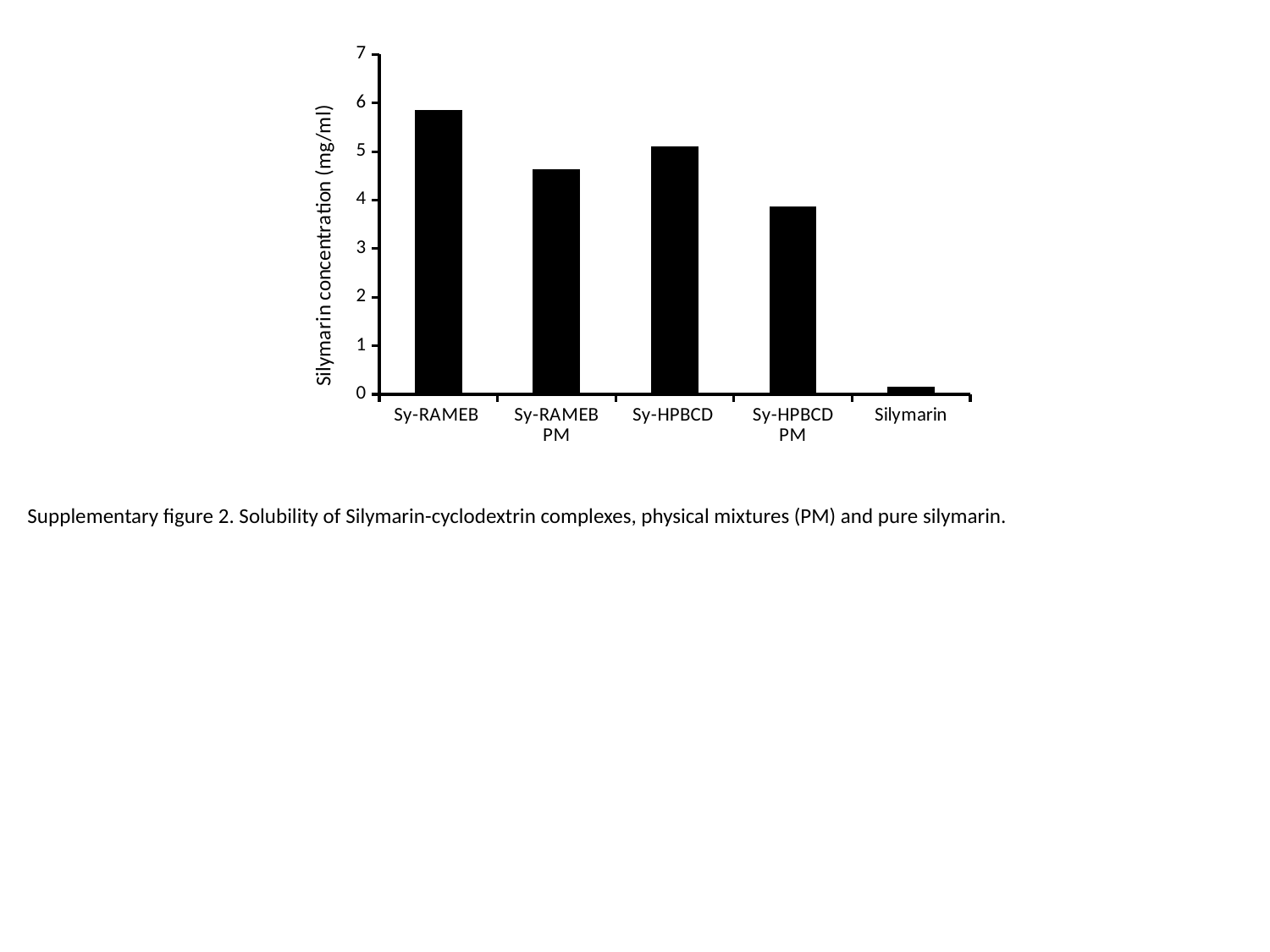

### Chart
| Category | |
|---|---|
| Sy-RAMEB | 5.862839879154079 |
| Sy-RAMEB PM | 4.636253776435046 |
| Sy-HPBCD | 5.101510574018128 |
| Sy-HPBCD PM | 3.862839879154079 |
| Silymarin | 0.15891238670694866 |Supplementary figure 2. Solubility of Silymarin-cyclodextrin complexes, physical mixtures (PM) and pure silymarin.
